# Buffalo Medical and Surgical Association

**Published:** 1886-09

**Authors:** 


### *BUFFALO MEDICAL AND SURGICAL ASSOCIATION.*

*Meeting of August* 3, 1886.

The President, Dr. W. W. Potter in the chair; Dr. F. R. Campbell, Secretary,

Present—Drs. Bartlett, Strong, Coakley, Runner, Howe, Clark, Brecht, Park, Wm. G. Ring, Hartwig, Hawkins, and, by invitation, Drs. Brush, of Philadelphia, and Miller

DR. F. W. BARTLETT read a paper, entitled "*Antiseptic Midwifery*," calling special attention to the use of turpentine as a germicide, to be used in the prophylaxis of puerpural fever.

DISCUSSION.

DR. STRONG had not used turpentine in his obstetric practice. He believed that 99 per cent. of the cases of puerperal fever occurred in patients where there had been incomplete contraction of the uterus. No physician has a right to leave the lying-in chamber until the uterus is thoroughly contracted. In his early practice he had had a number of fatal cases of puerperal septicæmia, and he believed that, with his present knowledge, all might have been saved.

Dr. Coakley stated that if, by any chance, he were to be limited in his practice to 15 or 20 remedies, he would retain turpentine in that number, as he considered it the most valuable antiseptic both for surgical and obstetrical purposes. He began the use of turpentine in midwifery upon Dr Bartlett's suggestion, and had been uniformly successful with it. In order to secure uterine contraction after delivery, he always gave ergot.

Dr. Clark had never used turpentine in midwifery, but considered it a valuable germicide. Used bi-chloride of mercury as an antiseptic, disagreed with Dr. Strong, who believed that septicæmia was usually due to insufficient contraction of uterus. Thought the essayist had been peculiarly fortunate in having but three lacerations of the perineum, in 220 cases of labor.

Dr. Howe spoke of importance of disinfecting the vagina before delivery, as advocated by Crede, in order to prevent ophthalmia neonatorum. Took issue with the essayist's hypothetical explanation of the germicidal properties of turpentine. It is easy to demonstrate the antiseptic properties of any drug. Exhibited a number of tubes containing germ cultivations and showed how to determine experimentally the antiseptic power of a drug. Stated that the bichloride and biniodide of mercury were the most powerful antiseptics.

Dr. Brush, during the past eight years, had delivered quite a number of insane women. Had always used antiseptics, with the best of results. He generally ordered the patient removed to a clean bed after delivery.

Dr. Hawkins had not used turpentine in obstetrical cases, and did not consider it necessary to use an antiseptic after normal delivery.

Dr. Hartwig thought Dr. Bartlett's use of turpentine was good as far as it went but something must, at times, be used per vaginum. Persulphate of iron he believed to be dangerous, and prefered salicylic acid, because it is not poisonous even in strong solutions. Thought that thorough uterine contraction would reduce the number of cases of puerperal fever.

Dr. W. W. Potter, criticised Dr. Coakley, who considered turpentine the most valuable antiseptic, showing that it ranked much lower than several others. He did not believe that there was such a thing as milk-fever, and considered these exacerbations of temperature "mild attempts at puerperal fever." Agreed with Dr. Howe in believing that antisepsis should extend to the child. Believed that a variety of disinfectants were valuable, to be selected according to the character of the cases. Advocated weak solutions of bichloride, 1 to 5,000. The odor of turpentine is offensive to many women.

Dr. Hartwig would not use salicylic acid, if puerperal fever were actually present.

Dr. Bartlett, in conclusion, stated that the relative value of an antiseptic determined experimentally, was a very different thing from the value determined clinically. The mercurial preparations may be the most powerful germicides but they have objections which preclude their use in many cases. Turpentine was most valuable as a prophylactic. It had never produced strangury in his practice.

*Voluntary Communications.*—Dr. Hartwig reported the case of a women afflicted with ascites. Paracentesis abdominis had been performed several times. Attempted continuous drainage into the subcutaneous tissues. Great œdema of the thighs and vulva resulted, and continued until the opening in the linea alba closed spontaneously. Abdominal tumors were detected by palpation before death. The autopsy revealed a contracted liver with perihepatic adhesions. There was hydro-nephrosis of both kidneys, the spleen was enlarged, with a few slight elevations externally. In the parenchyma of the spleen were found two tumors which were supposed to be of syphilitic origin.

*Prevailing Diseases.*—Diarrhœal affections.

*Special Meeting, August* 14, 1886.

The President, Dr. W. W. Potter in the chair; Dr. F. R. Campbell, Secretary.

Present—Drs. C. L. Dayton, Hauenstein, Ring, Bartlett, Rochester, Park, Folwell and Coakley.

The President, in opening the meeting, called attention to the fact that for the third time within a year the association was called upon to mourn the death of a distinguished member. Dr. Frank H. Hamilton of New York, who was for fifteen years a resident and practitioner in Buffalo, who was one of the founders of the Buffalo Medical and Surgical Association and of the Medical Department of the University of Buffalo, died on Wednesday, August 11th, thus ending a life which had reflected the greatest honor upon American medicine and upon the profession of this city, which was the field of some of his best labors and experience.

After remarks had been made by Drs. C. L. Dayton, Hauenstein and Bartlett, who had had professional relations with Dr. Hamilton, Dr. Rochester offered the following memorial which it was voted should be spread upon the minutes: "Frank Hastings Hamilton has finished his earthly career full of years and full of honors, and we cannot doubt that he has been called to receive the reward of a life well spent, thoroughly devoted to the comfort, welfare, and amelioration of his fellows. A long retrospect carries the speaker to the days when he was pupil, friend, colleague, and intimate associate of the departed, a period of forty years, and through it all a pleasant memory extends—a memory of assistance and advice—of many professional and social interviews, always enriched and illustrated by reminiscence or anecdote in a voice so eloquent, so persuasive, and so gentle that it was music in its every note. Those who have only heard Dr. Hamilton in recent years, since his vocal affection, can form no idea of the spell by which his utterance held the listener in the lecture-room and in debate. For over twenty years Buffalo was Dr. Hamilton's home, and that it was so in every sense of the word, by his professional success, by his host of warmly-attached friends, by his reputation extending from this, as a center, cannot be doubted, and we felt that indeed he was going "away from home" when he followed, within a year his colleague, the late Dr. Austin Flint, to the great city. He was attracted thither to found a new metropolitan medical college, to find a broader field for his usefulness, and to issue, with increased prestige, his new work on surgery, his "Fractures and Dislocations" having already been published and received in the most flattering manner. There is some question, in fact, whether this is not the most useful of any of his publications It contains a mine of statistical information, never before made public, gives the very best advice, and has saved many physicans from the persecutions and annoyances of malpractice suits, which it has done more than any other publication to stop. This work alone has made him an inestimable benefactor to the whole medical profession, and as such he will ever be recognized and remembered.

"In 1871, his voice having failed him, so that it was almost impossible for him to articulate, he was obliged to abandon didactic lecturing, in which he had been so strong and so attractive, but continued, for some years, his clinical illustrations and busied himself with his publications, his surgical history of the War, and general consultations. The most memorable of these was when, with Dr. Agnew, of Philadelphia, he was invited to see the late President Garfield, a call recognizing his eminent ability and skill. As an operator I do not think he had his superior. He was neat, graceful, and precise, and very fortunate. It is not necessary to enumerate the dates of Dr Hamilton's various positions and services; they are already mentioned in the daily journals. It is upon his private life that it is most pleasant to dwell. He was the very soul of chivalry. His reading was varied, and the poetry, of which he was so fond, gave a delicacy and refinement to his thoughts, which was constantly manifesting itself in his conversation. He always, when it was proper, sustained his professional brethern, and to the young he was especially cheering. Many a young practitioner, in the doubt and embarrassment of his early days of practice, has passed from his presence with cleared brow and buoyant hopes. I have said he was chivalrous. He was sometimes irritable, and occasionally, in the moment of excitement, would sometimes say or do something hasty, but he never bore ill-will, and often and often has hastened to take back an expression of annoyance, in the most frank and honorable manner He has gone to his rest Long will he be remembered most pleasantly and gratefully. He has not lived in vain."

